# Takayasu Arteritis Initially Mimicking Infective Endocarditis

**DOI:** 10.4084/MJHID.2011.040

**Published:** 2011-09-08

**Authors:** Aytekin Alcelik, Sevim Karacay, Ismail Necati Hakyemez, Busra Akin, Serkan Ozturk, Haluk Savli

**Affiliations:** Deparment of Internal Medicine, Abant Izzet Baysal University, Bolu, Turkey

## Abstract

Takayasu’s arteritis (TA) is a chronic inflammatory disease that affects large vessels, predominantly the aorta and its main branches, leading to vessel wall thickening, fibrosis and stenosis. Cardiac and vascular symptoms are also commonly present at disease onset. In TA with thoracic or abdominal involvement, although murmur can be present at physical examination, the pulse difference may be absent. Here, we report a case of TA who initially resembled infective endocarditis and had widespread thoracic murmur.

## Introduction:

Takayasu’s arteritis (TA) is a chronic inflammatory disease that affects large vessels, predominantly the aorta and its main branches, leading to vessel wall thickening, fibrosis and stenosis. The etiology and predisposing factors of TA are so far unknown. The main clinical symptoms and signs are weakness, fever, arthralgia, hypertension, intermittent claudication of the upper or lower limbs, cardiac diseases (cardiac failure, valvular or ischemic heart disease) and impaired renal function. Laboratory findings are non-specific. Treatment of the active disease is primarily based on corticosteroids.^1^–^2^

Cardiac and vascular symptoms are also commonly present at disease onset. In TA with thoracic or abdominal involvement, although murmur can be present at physical examination, the pulse difference may be absent.^3^–^4^ Therefore, TA can be misdiagnosed as infective endocarditis because of murmur and fever associated with the active phase of the disease.

Here, we report a case of TA who initially resembled infective endocarditis and had widespread thoracic murmur.

## Case:

**Figure 1.**
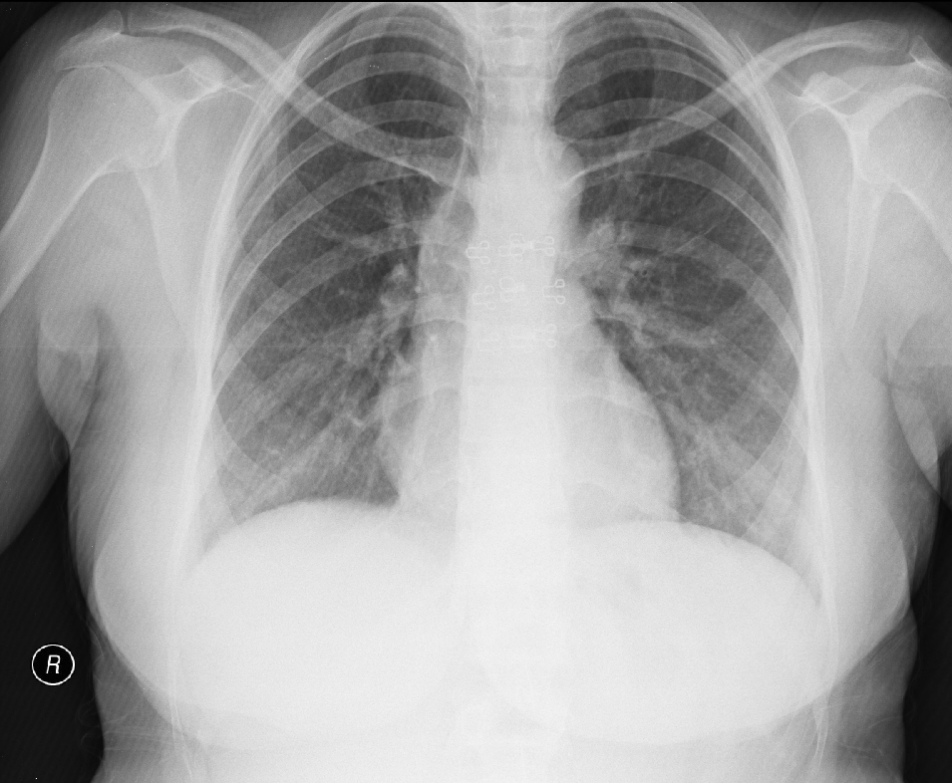
Telecardiography showing a normal heart and no pulmonary congestion.

A 30-year-old woman was admitted with the complaint of sore throat and polyarthralgia for five days. The patient also reported headache, dizziness and fever. At presentation, her body temperature was 37.5°C and blood pressure in right arm was 180/90 mmHg and in left arm was 170/80 mmHg. Physical examination showed widespread thoracic murmur and weak pulses in both arms. Ophthalmoscopic examination was normal. Laboratory findings were as follows: hemoglobin was 12.2 g/dL, hematocrit was 36%, white blood cell count was 12,500/mm^3^, platelet count was 296,000/mm^3^ and mean corpuscular volume was 80 femtoliter. Fasting glucose level, calcium, albumin, urea and creatinine levels were within the normal range. Sedimentation rate was 31 mm, C reactive protein (CRP) was 52 mg/dL (range from 0–3.19 mg/dL). Urine examination was normal. Throat culture did not yield beta hemolytic Streptococci. Telecardiography (Figure 1) and electrocardiogram were normal.

The patient had a preliminary diagnosis of infective endocarditis and was started on a treatment regimen consisting of ampicillin/sulbactam 4 x 1 g/day and gentamicin 1x160 mg/day. Echocardiography examination showed normal left cardiac functions, valves and intact interatrial septum. Doppler ultrasound showed kidneys of normal size. Serologic tests were negative for antinuclear antibody, VDRL, rheumatoid factor, hepatitis B and C, HIV and brucella. Blood and urine cultures were also negative.

**Figure 2.**
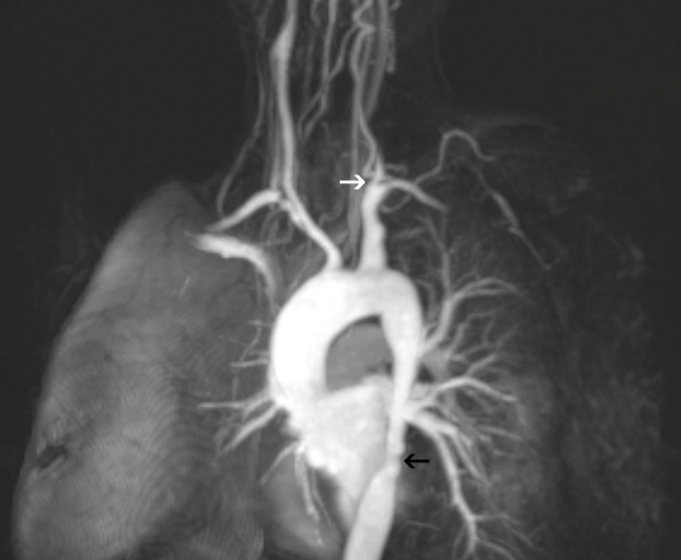
Magnetic resonance angiography showed minimal flow in the left common carotid artery (white arrow) and severe narrowing of the aortic thoracic segment (black arrow).

Patient was evaluated again because of sterile blood cultures and echocardiographic finding negative for infective endocarditis. Bilateral calf and upper limb claudicatio, Raynaud’s phenomenon, polyarthralgia and skin pale on both her forearms were also reported throughout in the previous year. Because of widespread thoracic murmur and hypertension, contrast-enhanced thoracic magnetic resonance angiography was performed. Magnetic resonance angiography showed minimal flow in the left common carotid artery, no flow in the homolateral subclavian artery and narrowing of the aortic thoracic segment to 10.4 mm (Figure 2).

Based on these findings, the patient was diagnosed with TA. Treatment with prednisolone at a dose of 48 mg/day was initiated.

## Discussion:

Bacterial endocarditis may present itself with rheumatological symptoms in 28–42% of patients .^5^ TA can be confused with infective endocarditis when ascending aorta and aortic valve are affected. As seen in our case, the widespread murmur occurring as a result of narrowing of the thoracic segment of aorta can mimic myocarditis or infective endocarditis. It must be kept in mind that, when the narrowing segment is limited to the distal part of the descending aorta, pulse difference may be absent. Some diseases complicating TA have been reported in the literature. TA can complicate Crohn’s disease, systemic lupus erythematosus, rheumatoid arthritis, Wegener’s granulomatosis and sarcoidosis.^6^–^8^ In the literature, no data was found on the association of TA and infective endocarditis. Clinically similar signs can be present in the active disease state.

TA has several important complications that determine the prognosis, including secondary hypertension, aortic regurgitation, aneurysms and retinopathy.^1^,^4^ Among these complications, only hypertension was found in our patient and was controlled well with 5 mg amlodipine. Progress in medical technology has made the diagnose of TA easier. Computed tomography and magnetic resonance imaging can help to detect vascular diseases.^9^ In our case, MRI angiography was useful in evaluating the arterial involvement in TA. To diagnose TA accurately and as promptly as possible, and to evaluate the disease appropriately, the combined use of various imaging techniques is necessary.

Depending on the affected vessels, TA may present a wide variety of clinical symptoms. In TA, if the the distal part of the aorta is narrowed, widespread thoracic murmur can be found in the absence of a pulse difference. TA must be considered one of the underlying diseases that may cause widespread thoracic systolic murmur in young women with normal echocardiography.
